# Human antibody V_H_ domains targeting uPAR as candidate therapeutics for cancers

**DOI:** 10.3389/fonc.2023.1194972

**Published:** 2023-10-09

**Authors:** Xiaojie Chu, Wei Li, Margaret G. Hines, Ilya Lyakhov, John W. Mellors, Dimiter S. Dimitrov

**Affiliations:** ^1^ Center for Antibody Therapeutics, Division of Infectious Diseases, Department of Medicine, University of Pittsburgh Medical School, Pittsburgh, PA, United States; ^2^ Comp IL, LLC, Carnegie, PA, United States; ^3^ Abound Bio, Pittsburgh, PA, United States

**Keywords:** therapeutic antibody, VH domain, human uPAR, DbTE (domain based bispecific T cell engager), cancer

## Abstract

The high expression of uPAR has been linked to tumor progression, invasion, and metastasis in several types of cancer. Such overexpression of uPAR makes it a potential target for immunotherapies across common cancers such as breast, colorectal, lung, ovarian cancer, and melanoma. In our study, two high-affinity and specific human V_H_ domain antibody candidates, designed as clones 3 and 115, were isolated from a phage-displayed human V_H_ antibody library. Domain-based bispecific T- cell engagers (DbTE) based on these two antibodies exhibited potent killing of uPAR-positive cancer cells. Thus, these two anti-uPAR domain antibodies are promising candidates for treating uPAR positive cancers.

## 1 Introduction

Urokinase-type plasminogen activator receptor (uPAR), also named CD87, is a single-chain membrane glycoprotein receptor containing three homologous domains (D1, D2, and D3) anchored to the cell membrane by a GPI linkage (1). In normal physiological conditions, uPAR expression is fairly low. This expression, however, can be highly elevated in many types of cancer including breast cancer (2), colorectal cancer (3), melanoma (4, 5), brain cancer (6), lung cancer (7), ovarian cancer (8), prostate cancer (9), liver cancer (10), gastric cancer (11), and pancreatic cancer (12). uPAR also plays an important role in tumor proliferation, metastasis, angiogenesis, and prognosis (13). Many studies have revealed that high expression of uPAR is related to poor prognosis and that expression level can serve as a marker of tumor malignancy (14, 15). Even though several systems are involved, the uPA-uPAR signaling pathway plays a key role from tumor proliferation to metastasis (16).

The uPA-uPAR-α5β1 integrin complex can bind to G-protein-coupled receptors (GPCRs) or interact with EGFR or PGGFRβ to activate focal adhesion kinase (FAK)-MAPK-ERK pathway and PI3K/AKT pathway, which promotes tumor cell proliferation and survival (17–20). uPAR’s expression on the non-malignant cells that infiltrate cancers and on malignant tumor cells adds to its importance in tumor progression and poor prognosis. When uPA is activated after binding to uPAR, plasminogen is cleaved into active plasmin, which further activates MMPs to degrade ECM and regulate cell migration (21, 22). Moreover, uPAR interacts with VEGFR2 and promotes VEGFR2 internalization, thus enhancing the VEGF-induced angiogenesis (23, 24). Taken together, uPAR’s expression in cancer and importance in tumor make the receptor an attractive therapeutic target for cancer treatment, in addition to prognosis and diagnosis.

The development of monoclonal antibody-based immunotherapy has opened new avenues to specifically target cancer cells expressing certain receptors. Such therapies have become an increasingly attractive option for cancer treatment due to its high efficacy and lower side effects compared to other options such as surgery, radiation, and chemotherapy. In addition to the binding to targeted antigens, antibodies can also mobilize anti-tumor immunity through effector functions. Different antibody structures have been developed in recent decades, including fragment antigen-binding region (Fab), single-chain Fv (scFv) fragments, and domain antibodies (V_H_ and V_L_), for tailored applications. Among these formats, antibody heavy chain variable (V_H_) domains have shown increasing promise in antibody-based cancer immunotherapy due to their small size (ranging from 11kDa to 15 kDa), high affinity, high yields, and low immunogenicity. Studies have shown that these lower molecular weight proteins can deeply penetrate tissues, and enabling immunotherapies to target new epitopes that are not accessible to large antibody constructs (25). Thus, the use of variable domain antibodies may be a powerful prove useful in the development of cancer immunotherapies, especially for solid tumors.

In our current study, we identified two potent human V_H_ domain antibodies that target human uPAR. These binders were characterized for their affinity and specificity. The domain-based bispecific T cell engager (DbTE) based on these two binders showed potent killing effects of uPAR-expressing cancer cells. To our knowledge, this is the first report of uPAR-specific human V_H_ domain antibodies as candidates for cancer immunotherapy.

## 2 Materials and methods

### 2.1 Panning of high- affinity V_H_ domains against uPAR from large V_H_ phage library

Human uPAR-Fc (Catalog # 10378-UK-100) and uPAR-His (Catalog # 807UK100CF) recombinant proteins were purchased from R&D system. To pan antibody candidates against uPAR, a 10^12^ large phage-displayed human immunoglobulin heavy chain variable domain (V_H_) library (26, 27) was used against human IgG1 Fc fused recombinant uPAR. The panning was performed as previously described (28–30). Briefly, the library were first blocked with 5% milk then incubated with uPAR-Fc following with Protein G magnetic beads (Thermo Fisher). The separated antigen-bound phages were then infected with TG1 for phages expression and amplification. After the first round panning against 5 μg uPAR-Fc, two additional rounds of panning were performed by using consecutively one-fold reduced antigen in each round to increase selective rigidity. 192 individual clones obtained from the final round of panning were screened for binding to uPAR-His protein by ELISA.

### 2.2 Expression and purification of V_H_, V_H_-Fc, and DbTE

To convert V_H_ antibody candidates to V_H_-Fc format, the V_H_ domain was amplified and cloned into the pcDNA-IgG1 Fc vector. For the construction of DbTE, humanized OKT3 scFv (VH-(G_4_S)_6_-VL) was inserted at the C terminal of V_H_ followed by the IgG1 Fc with LALAPG mutation. The expression and purification were performed as previously described (28). Both the V_H_-Fc and DbTE were transiently transfected and expressed by the Expi293 expression system, then purified by protein A resin (Thermo Fisher). The V_H_ binder was expressed in E.coli TopF expression system and purified on Ni-NTA columns (GE Healthcare).

### 2.3 ELISA

The binding and specificity of V_H_, V_H_-Fc, and DbTE to uPAR or CD3 were analyzed by ELISA. uPAR-His protein or CD3 protein was coated at 50 ng/well at 4°C overnight, then blocked with 5% milk for 1 hours at 37°C. After washing 3 times by 0.05% PBST, 3-fold serially diluted V_H_ and V_H_-Fc binders were incubated on the plate for 1 hour at 37°C. The binding of V_H_ candidates was detected by anti-FLAG M2-peroxidase (HRP) antibody (Sigma-Aldrich) while V_H_-Fc or DbTE binding was detected by HRP conjugated goat anti-human IgG1 Fc (Sigma-Aldrich) at 1:1000 dilution. The plates were washed 3 times by 0.05% PBST between each reagents incubation. Binding activity was detected using 3,3′,5,5′-tetramethylbenzidine (Sigma-Aldrich) and was stopped by TMB stop buffer (ScyTek Laboratories). Absorbance was read at 450 nm.

### 2.4 BLItz

DPBS was used to establish a baseline for 30s. Streptavidin biosensors (ForteBio) were coated with 16.7 μg/mL recombinant uPAR-Biotin for 2 min. For competition assay, 500nM of V_H_ 3 were used for association and monitored for 2 min, then 500nM of V_H_ 115 were used for continuing association and monitored for 2 min. For affinity assay, 400nM, 200nM and 100nM of V_H_, V_H_-Fc, and DbTE were used separately for association and monitored for 2min. Dissociation was monitored in DPBS for 4 min.

### 2.5 Size exclusion chromatography

The aggregation of the antibodies were analyzed by Superdex 200 Increase 10/300 GL chromatography (GE Healthcare, Chicago, IL, USA) as previously described (28). 200 μg of filtered antibodies were analyzed and eluted by DPBS buffer at a flow rate of 0.5 mL/min.

### 2.6 Cells

Expi293 cells (Thermo Fisher) were maintained in an Expi293 expression medium supplemented with 0.4% penicillin-streptomycin (P/S). 293T cells and A375 human melanoma cells were purchased from ATCC and were maintained in DMEM medium supplemented with 10% FBS and 1% P/S separately. T cells were isolated from healthy donor’s PBMCs (Zen-Bio) by using the human Pan T cell isolation kit (Miltenyi Biotec) and activated by CD3/CD28 T cell activator Dyna beads (Gibco) at 1:1 cell-bead ratio for 48 h. The activated T cells were used for the cytotoxicity assay of the DbTE antibody.

### 2.7 Flow cytometry

The cell surface expression level of uPAR protein was detected by a commercial antibody. 2 ×10^5^ cells/test were stained with mouse anti-human uPAR antibody (R&D systems, Catalog# MAB807) or an isotype antibody for 30 min at 4 °C followed by PE-conjugated anti-mouse IgG secondary antibody. To verify the cell surface binding of the isolated antibody, cells were incubated with V_H_-Fc 3 or V_H_-Fc 115 at a concentration of 50 nM, or V_H_ 3 or V_H_ 115 at a concentration of 1 µM for 30 min at 4 °C. Cells were then stained with a secondary antibody, goat anti-human IgG (γ-chain specific)-PE (Sigma-Aldrich, 1:250, Catalog# P9170) for V_H_-Fc or anti-Flag-APC (Miltenyi Biotec, Catalog#130-119-584) for V_H_. An irrelevant V_H_-Fc and V_H_ were used as isotype controls.

### 2.8 Cytotoxicity assays

The cell cytotoxicity of anti-uPAR DbTE was measured by LDH-Glo cytotoxicity assay kit (Promega) following the manufacturer’s instructions. Target cells (1 ×10^4^ cells/well) and activated T cells were seeded in a 96-well plate at an E: T ratio 10:1, mixed with serially diluted DbTE antibodies in a growth medium, and incubated for 24h at 37°C in 5% CO_2_ humidified atmosphere. The final volume was 100 μl/well. The cell supernatant was diluted 20-fold and incubated for 50 min for LDH assay setup. The calculation of relative % cytotoxicity is as follows: relative % cytotoxicity = 100 × (Experimental LDH release – Target and effector cell only)/(maximum LDH release control – Background).

### 2.9 Statistical analysis

Statistical analyses were performed by GraphPad Prism. Differences were considered statistically significant when *p*< 0.05. Significance was tested using two-way ANOVA, followed by Tukey’s multiple comparisons tests. ****, *p*<0.0001.

## 3 Results

### 3.1 Selection and characterization of high-affinity V_H_ antibodies against human uPAR

**Figure 1:**
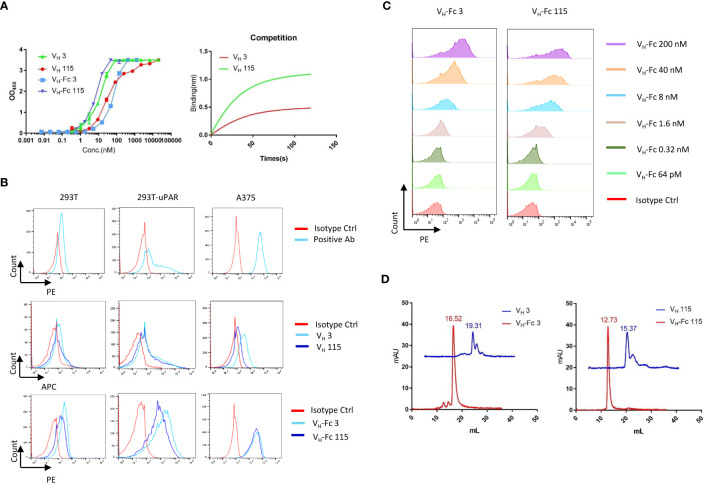
Specificity of V_H_/V_H_-Fc with human uPAR on cell surface **(A)** Anti-uPAR V_H_ and V_H_-Fc binding to recombinant human uPAR measured by ELISA (left), and competition BLItz of V_H_ 3 with V_H_ 115 at 500nM for binding to uPAR (right); **(B)** Cell surface detection of uPAR by commercial mouse anti-uPAR antibody, V_H_ 3 and 115 (1μM), and V_H_-Fc 3 and 115 (50nM) on 293T, 293T-uPAR, and A375 cells measured by Flow cytometry; **(C)** Dose-dependent cell surface binding of V_H_-Fc 3 and 115 on 293T-uPAR cells. **(D)** Aggregation evaluation of V_H_ 3 and V_H_-Fc 3 (left), V_H_ 115 and V_H_-Fc 115 (right) measured by SEC.

A large phage-displayed human V_H_ library was used to pan against recombinant human uPAR protein for antibody selection. Several V_H_ binders were identified after three rounds of panning. Two antibodies, designated as V_H_ 3 and V_H_ 115, were selected based on their high affinity, specificity, and other desirable properties. The amino acid sequence of these two binders are shown in **Table 1**. The EC_50_ values of V_H_ 3 and V_H_ 115 as determined by ELISA were 12.1 ± 0.8nM and 34.2 ± 3.5 nM, respectively (**Figure 1A**). The equilibrium dissociation constant (K_D_) values were 17.1 nM and 1.7 nM respectively as determined by BLItz (**Table 2**). Additionally, these two binders did not bind to BSA at high concentrations of 66.7 μM, and their binding affinity are not affected in the presence of serum, indicating their specificity for uPAR (data not shown). The competition BLItz experiment showed that V_H_ 3 and V_H_ 115 target different binding epitopes on human uPAR (**Figure 1A**). To increase the binders’ half-life and avidity, the two V_H_ binders were converted to V_H_-Fc format by fusing IgG1 Fc into the C-terminal of V_H_. The EC_50_ values of V_H_-Fc 3 and V_H_-Fc 115 as detected by ELISA were 64.3 ± 2.3 nM and 6.6 ± 0.2 nM, respectively (**Figure 1A**). The KD values were 9.6 nM and 71.1 nM, respectively as determined by BLItz (**Table 2**). To verify the specificity of these binders to uPAR expressed on the cell surface, the surface expression of uPAR on parental 293T cells, 293T cells isogenically expressing uPAR (293T-uPAR), or A375 cells (human melanoma cell line intrinsically express uPAR) were verified by a commercial anti-human uPAR antibody. Among these cells, 293T cells (MFI of isotype vs positive uPAR Ab is 62 vs 126) showed a low expression of uPAR while 293T-uPAR cells (MFI of isotype vs positive uPAR Ab is 46.4 vs 176) and A375 cells (MFI of isotype vs positive uPAR Ab is 71.3 vs 4775) showed a high expression level of uPAR (**Figure 1B**). Next, the binding specificity of our newly identified binders was tested on the above cell lines. The two V_H_ and V_H_-Fc binders showed a high binding to both 293T-uPAR and A375 cells, while a low level of binding to 293T cells (**Figure 1B**). These results were consistent with the expression level of uPAR on these cell lines. Moreover, both V_H_-Fc binders bound to the 293T-uPAR cells in a concentration-dependent manner and the estimated on-cell binding avidity of V_H_-Fc 3 and V_H_-Fc 115 were 43.1nM and 10.3nM, respectively (**Figure 1C**). Protein folding was assessed by the size-exclusion chromatography (SEC). Based on the molecular weight calibration curves, while V_H_ 3 and V_H_-Fc 3 exhibit monomeric folding, V_H_ 115 and V_H_-Fc 115 both showed a dimeric folding (**Figure 1D**). The late-elution peaks may be due to VH interaction with the column agarose matrix. The stability of these VH domains are further enhanced after converting to the VH-Fc format. We found that the VH-Fc proteins exhibit homogenous folding peaks. The Fc fragments may help to stabilize the VH domain.

**Table 1:** Amino acid sequence of human uPAR antibodies.

Antibody	Amino Acid Sequence
V_H_ 3	EVQLVESGGGLVQPGGSLRLSCAASGFTFSRYWMSWVRQAPGKALEWIGEINHSGSTNYNPSLKSLVTISRDNSKNTLYLQMNSLRAEDTATYYCARSLV PALSYYYYYGMDVWGQGTTVTVSS
V_H_ 115	EVQLVESGGGLVQPGGSLRLSCKGSGFTFGDYAIGWVRQAPGQRLEWIGWINTNSGSPKYAQGFTGRFTISRDNSKNTLYLQMNSLRAEDTAVYYCATD VVVPWGQGSQVTVSS

**Table 2:** BLItz results of human uPAR antibodies.

Antibody	k_on_ (M^−1^s^−1^)^1^	k_off_ (s^−1^)^1^	K_D_ (nM)^1^
V_H_ 3	7.9 × 10^4^ ± 5.7 × 10^2^	1.4 × 10^-3^ ± 1.9 × 10^-5^	17.1
V_H_-Fc 3	1.2 × 10^5^ ± 2.3 × 10^3^	1.2 × 10^-3^ ± 4.8 × 10^-5^	9.6
DbTE 3	1.1 × 10^5^ ± 1.5 × 10^3^	7.2 × 10^-4^ ± 2.7 × 10^-5^	6.6
V_H_ 115	2.1 × 10^4^ ± 2.9 × 10^2^	3.6 × 10^-5^ ± 1.3 × 10^-5^	1.7
V_H_-Fc 115	3.3 × 10^4^ ± 1.5 × 10^3^	2.4 × 10^-3^ ± 5.6 × 10^-5^	71.1
DbTE 115	3.6 × 10^4^ ± 4.1 × 10^2^	4.2 × 10^-5^ ± 1.6 × 10^-5^	1.2

^1^Mean kinetic rate constants (k_on,_ k_off_) and equilibrium dissociation constants (K_D_ = k_off_/k_on_) were determined from curve fitting analyses of BLItz results.

### 3.2 V_H_ domains-based T cell engagers (DbTEs) show potent cytotoxicity against uPAR expressing cells

**Figure 2:**
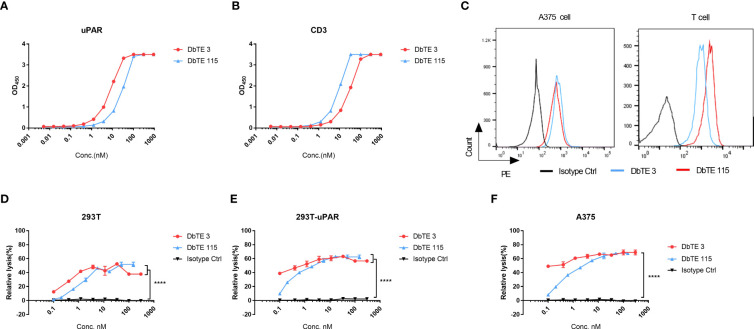
*In vitro* cytotoxicity of T cells to uPAR-expressing cells by anti-uPAR DbTE. **(A, B)** DbTE 3 and 115 binding to uPAR **(A)** and CD3 **(B)** measured by ELISA. **(C)** Cell binding of 100nM DbTE 3 and 115 on A375 cells and T cells tested by flow cytometry. **(D-F)** Percent relative lysis of 293T cells **(D)**, 293T-uPAR cells **(E)**, and A375 cells **(F)** by T cells mediated by DbTE 3 and 115, respectively. T cells and target cells were added at E: T ratio of 10:1 and simultaneously treated with serially diluted DbTE antibodies for 24h. Experiment was repeated two times. Values were reported as the mean of percent relative lysis ± SD. Significance was tested by using two-way ANOVA, followed by Tukey’s multiple comparisons test. ****p<0.0001.

As a proof of concept, we generated and assessed the cell cytotoxicity of anti-uPAR domain antibody-based bispecific T cell engagers (DbTEs) against uPAR expressing cancer cells. To construct DbTE, we fused VH domains to the humanized anti-CD3 antibody OKT3 scFv, which is in frame to the human IgG1 Fc with FcγR binding silencing mutations (LALAPG). The EC_50_ of DbTE 3 and 115 for binding to the recombinant human uPAR protein as tested by ELISA were 7.7 ± 0.4 nM and 28 ± 2.1 nM, respectively (**Figure 2A**). The EC_50_ of DbTE 3 and 115 for binding to the human CD3 protein as tested by ELISA were 28.8 ± 1.8 nM and 8.7 ± 0.6 nM, respectively (**Figure 2B**). The KD values of DbTE 3 and 115 were 6.6 nM and 1.2 nM, respectively (**Table 2**). DbTE binding to A375 tumor cells and T cells was verified by flow cytometry (**Figure 2C**). Next, T- cell-mediated cytotoxicity against uPAR positive cancer cells induced by each DbTE was assessed using an LDH assay. Dose-dependent lysis was observed at the E: T ratio of 10:1 on 293T (**Figure 2D**), 293T-uPAR cells (**Figure 2E**), and A375 cells (**Figure 2F**) mediated by DbTE 3 or DbTE 115. A lower level of lysis was observed with 293T cells, consistent its lower level of uPAR on the cell surface. Moreover, DbTE 3 appear to be more effective than DbTE 115 at low concentrations. The estimated cell killing IC_50_ of DbTE 3 and 115 on 293T cells were 0.3 ± 0.3 nM and 1.1nM ± 0.3 nM respectively, on 293T-uPAR cells were 0.07 ± 0.6 nM and 0.4 ± 0.2 nM respectively, and on A375 cells were 0.06 ± 0.5 nM and 0.5 ± 0.2 nM respectively.

## 4 Discussion

uPAR is a glycoprotein receptor that is highly expressed in many solid cancers including breast, lung, prostate, ovarian, and liver cancer. Moreover, uPAR is also highly expressed on stromal cells in the tumor microenvironment, such as vascular endothelial cells, tumor-related fibroblasts, and macrophages. The multifunctionality of uPAR ranging from tumor progression, invasion, and angiogenesis to metastasis makes it an ideal target for cancer therapy. Currently, several antibody-based therapies targeting uPAR performed in preclinical showed promising effects in breast cancer (31, 32) but there have no antibody-based therapies targeting uPAR in clinical trials yet. However, several diagnostic clinical trials detecting uPAR for cancer and metastasis have demonstrate safe and clinical potential (33–35). Hence, characterization of novel antibodies with diverse affinity, specificity, and size may be useful in the treatment of cancers with high uPAR expression.

In our study, we selected and characterized two fully human V_H_ domain antibodies that target uPAR. Both antibodies showed high affinity for uPAR. By analyzing the sequence using IMGT/DomainGapAlign, the V_H_ 3 and 115 showed 81.6% and 78.4% identity with IGHV 3-23*04 germline, separately. The CDR-IMGT lengths of V_H_ 3 and V_H_ 115 are [8.7.18] and [8.8.7]. Converting the antibodies to V_H_-Fc fusion protein enhanced the avidity of V_H_ 3 by 2-fold, but decreased avidity 40-fold in V_H_ 115 (**Figure 1A**). This decrease may be due to the aggregation. Further antibody maturation of V_H_ 115 to decrease the aggregation is needed. The killing effects of DbTEs based on these two antibodies showed specific cell killing on cells with observable expression levels of uPAR, demonstrating their potential for cancer immunotherapies. Lower killing effects were observed when targeting 293T cells with lower uPAR expression levels compared with the 293T-uPAR overexpressing cell line (**Figures 2D, E**). These findings warrant further characterization of these antibodies’ specificity, efficacy, and toxicity as well as comparison with other uPAR IgG antibodies for cancer inhibition *in vivo* and the application potential of domain antibodies. Moreover, further investigation is needed to observe the ability of these antibodies to block the uPAR-uPA signaling pathway.

Recent studies have also shown that uPAR is associated with senescence-associated pathologies (36, 37). Further development of these antibodies as senolytic reagents may increase their potential for treating senescence-related diseases, such as fibrin-associated inflammation and liver fibrosis (38). In summary, the anti-uPAR antibodies described above showed significant potential in heavy chain variable domain antibody-based immunotherapies and may be useful in targeting diseases related to the elevated expression level of uPAR.

## Data availability statement

The original contributions presented in the study are included in the article/supplementary material. Further inquiries can be directed to the corresponding authors.

## Ethics statement

Ethical approval was not required for the studies on humans in accordance with the local legislation and institutional requirements because only commercially available established cell lines were used.

## Author contributions

DD, JM, and XC conceived and designed the research. XC identified and characterized antibodies, designed and performed functions assays. WL made V_H_ phage libraries. XC wrote the draft of the article. WL, MH, IL, JM, and DD revised the manuscript. All authors discussed the results and contributed to the final manuscript.
